# Supplementary material for: A Radiogenomics Ensemble to Predict EGFR and KRAS Mutations in NSCLC
Source: Tomography. 2021 Apr 29;7(2):154–68. doi: 10.3390/tomography7020014 (PMC8162978; doi:10.3390/tomography7020014)
Supplement: Supplementary file 1 [file tomography-07-00014-s001.zip › Clinical Data Train and Test sets.pdf]

Table S1. Clinical Variables Training Dataset

Variable	Overall Dataset	EGFR Mutant	EGFR Wildtype	KRAS Mutant	KRAS Wildtype
Median Age (Range)	69<br>46 - 85	71<br>62 - 85	69<br>46-84	68<br>50 - 81	69<br>46 - 85
Gender					
Male<br>Female	59 (83%)<br>12 (17%)	6 (8%)<br>3 (4%)	53 (75%)<br>9 (13%)	14 (20%)<br>2 (3%)	45 (63%)<br>10 (14%)
Smoking Status					
Current<br>Former<br>Non-smoker	16 (23%)<br>47 (66%)<br>8 (11%)	1 (1%)<br>6 (8%)<br>2 (3%)	15 (22%)<br>41 (58%)<br>6 (8%)	6 (8%)<br>10 (14%)<br>0 (0%)	10 (15%)<br>37 (52%)<br>8 (11%)
Pathological T Stage					
Tis<br>T1a<br>T1b<br>T2a<br>T2b<br>T3<br>T4	3 (4%)<br>16 (23%)<br>16 (23%)<br>23 (32%)<br>3 (4%)<br>6 (8%)<br>4 (6%)	1 (1%)<br>1 (1%)<br>3 (4%)<br>3 (4%)<br>0 (0%)<br>1 (1%)<br>0 (0%)	2 (3%)<br>15 (22%)<br>13 (19%)<br>20 (28%)<br>3 (4%)<br>5 (7%)<br>4 (6%)	0 (0%)<br>4 (6%)<br>3 (4%)<br>5 (7%)<br>1 (1%)<br>3 (4%)<br>0 (0%)	3 (4%)<br>12 (17%)<br>13 (19%)<br>18 (25%)<br>2 (3%)<br>3 (4%)<br>4 (6%)
Pathological N Stage					
N0<br>N1<br>N2	54 (76%)<br>8 (11%)<br>9 (13%)	8 (11%)<br>1 (1%)<br>0 (0%)	46 (65%)<br>7 (10%)<br>9 (13%)	12 (17%)<br>1 (1%)<br>3 (4%)	42 (59%)<br>7 (10%)<br>6 (9%)
Pathological M Stage					
M0<br>M1b	69 (97%)<br>2 (3%)	9 (13%)<br>0 (0%)	60 (84%)<br>2 (3%)	15 (22%)<br>1 (1%)	54 (75%)<br>1 (2%)
Histology					
Adenocarcinoma<br>Squamous cell carcinoma<br>NSCLC NOS	54 (76%)<br>14 (20%)<br>3 (4%)	9 (13%)<br>0 (0%)<br>0 (0%)	45 (63%)<br>14 (20%)<br>3 (4%)	15 (22%)<br>0 (0%)<br>1 (1%)	39 (54%)<br>14 (20%)<br>2 (3%)

Table S2. Clinical Variables Test Dataset

Variable	Overall Dataset	EGFR Mutant	EGFR Wildtype	KRAS Mutant	KRAS Wildtype
Median Age (Range)	67<br>50 - 81	79<br>55 - 81	65<br>50 - 81	67<br>65 - 79	65<br>55 - 81
Gender					
Male<br>Female	6 (50%)<br>6 (50%)	1 (8%)<br>2 (17%)	5 (42%)<br>4 (33%)	3 (25%)<br>1 (8%)	3 (25%)<br>5 (42%)
Smoking Status					
Current<br>Former<br>Non-smoker	2 (17%)<br>9 (75%)<br>1 (8%)	0 (0%)<br>2 (17%)<br>1 (8%)	2 (17%)<br>7 (58%)<br>0 (0%)	0 (0%)<br>4 (33%)<br>0 (0%)	2 (17%)<br>5 (42%)<br>1 (8%)
Pathological T Stage					
Tis<br>T1a<br>T1b<br>T2a<br>T2b<br>T3<br>T4	0 (0%)<br>1 (8%)<br>3 (25%)<br>3 (25%)<br>3 (25%)<br>2 (17%)<br>0 (0%)	0 (0%)<br>0 (0%)<br>2 (17%)<br>0 (0%)<br>1 (8%)<br>0 (0%)<br>0 (0%)	0 (0%)<br>1 (8%)<br>1 (8%)<br>3 (25%)<br>2 (17%)<br>2 (17%)<br>0 (0%)	0 (0%)<br>0 (0%)<br>0 (0%)<br>2 (17%)<br>0 (0%)<br>2 (17%)<br>0 (0%)	0 (0%)<br>1 (8%)<br>3 (25%)<br>1 (8%)<br>3 (25%)<br>0 (0%)<br>0 (0%)
Pathological N Stage					
N0<br>N1<br>N2	11 (92%)<br>0 (0%)<br>1 (8%)	2 (17%)<br>0 (0%)<br>1 (8%)	9 (75%)<br>0 (0%)<br>0 (0%)	4 (34%)<br>0 (0%)<br>0 (0%)	7 (58%)<br>0 (0%)<br>1 (8%)
Pathological M Stage					
M0<br>M1b	11 (92%)<br>1 (8%)	3 (25%)<br>0 (0%)	8 (67%)<br>1 (8%)	4 (34%)<br>0 (0%)	7 (58%)<br>1 (8%)
Histology					
Adenocarcinoma<br>Squamous cell carcinoma<br>NSCLC NOS	12 (100%)<br>0 (0%)<br>0 (0%)	12 (100%)<br>0 (0%)<br>0 (0%)	12 (100%)<br>0 (0%)<br>0 (0%)	12 (100%)<br>0 (0%)<br>0 (0%)	12 (100%)<br>0 (0%)<br>0 (0%)
