# Supplementary material for: A Radiogenomics Ensemble to Predict EGFR and KRAS Mutations in NSCLC
Source: Tomography. 2021 Apr 29;7(2):154–68. doi: 10.3390/tomography7020014 (PMC8162978; doi:10.3390/tomography7020014)
Supplement: Supplementary file 1 [file tomography-07-00014-s001.zip › CNN ARCHITECTURES.pdf]

Architecture 1

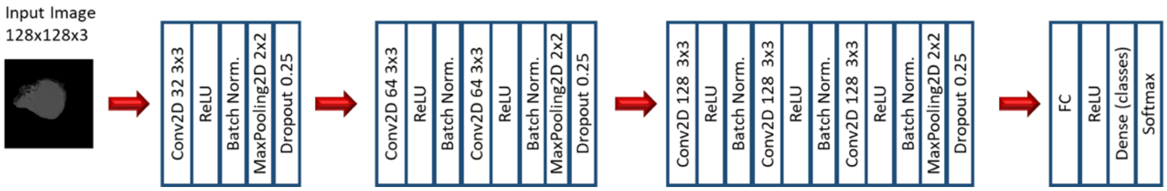

Architecture 2

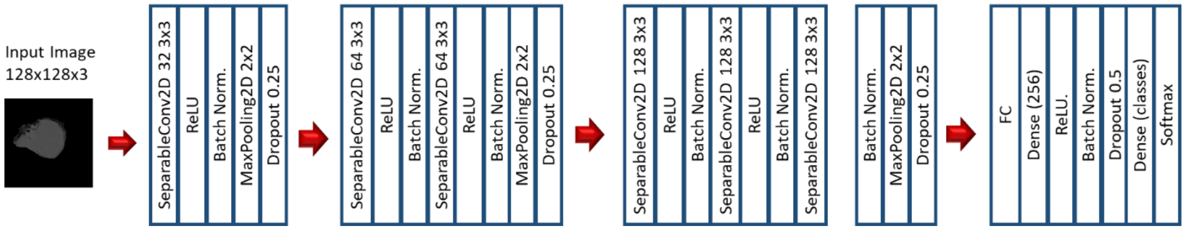

Architecture 3

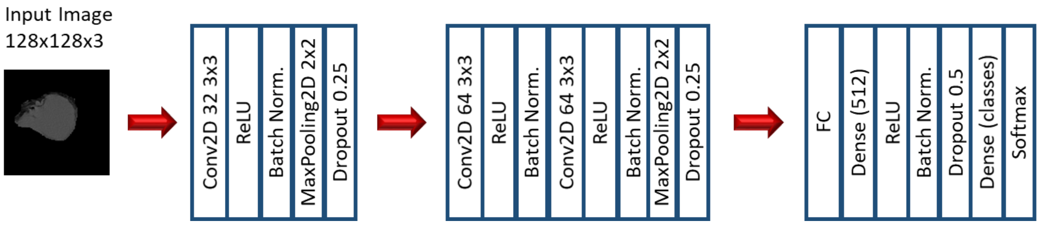

Architecture 4

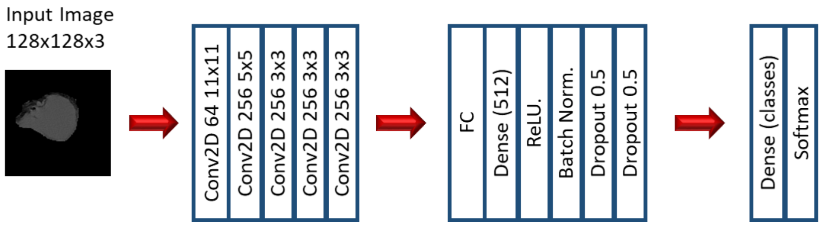

Architecture 5

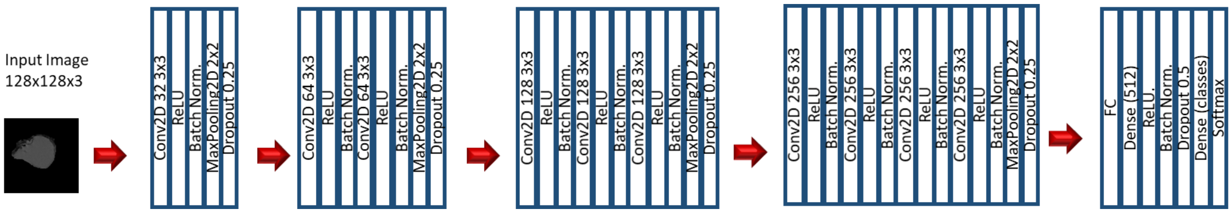

Architecture 6

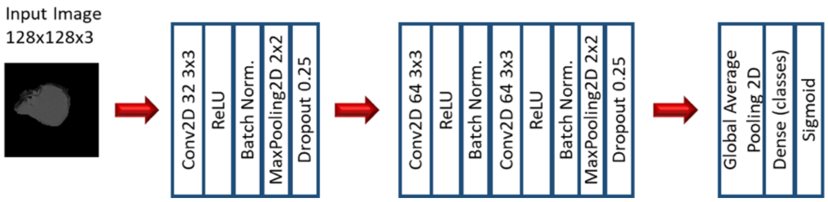

Architecture 7

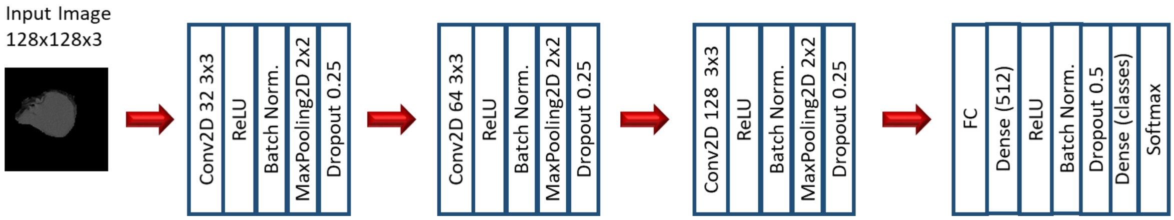

Architecture 8

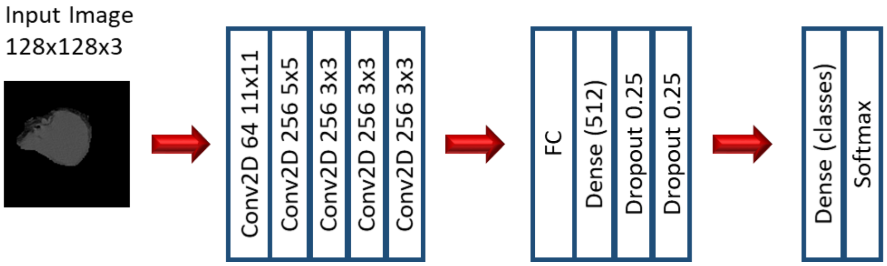

Architecture 9

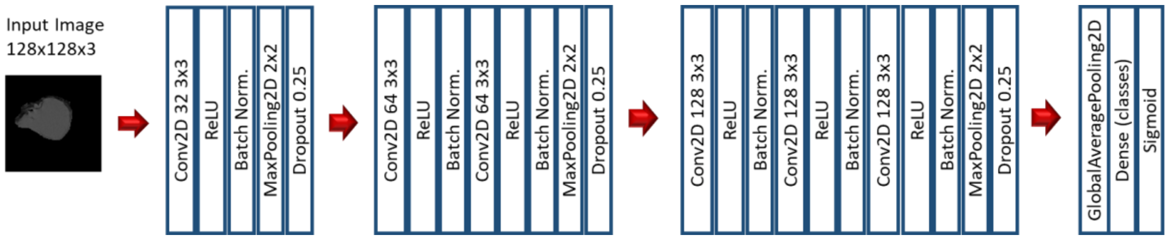

Architecture 10

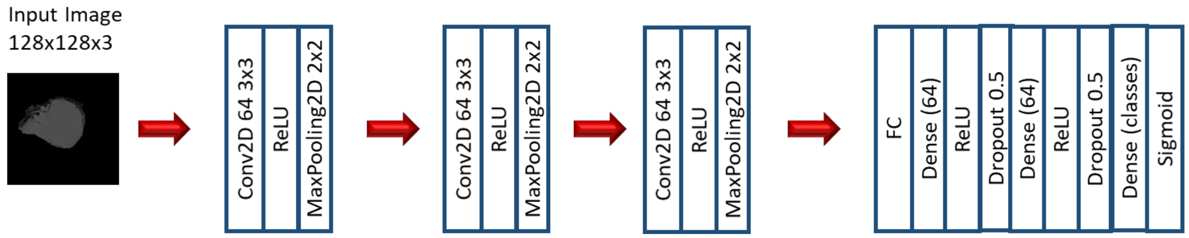

Figure S1 CNN ARCHITECTURES
