# Supplementary material for: A Radiogenomics Ensemble to Predict EGFR and KRAS Mutations in NSCLC
Source: Tomography. 2021 Apr 29;7(2):154–68. doi: 10.3390/tomography7020014 (PMC8162978; doi:10.3390/tomography7020014)
Supplement: Supplementary file 1 [file tomography-07-00014-s001.zip › Results Train and Test.pdf]

Results Train and Test

Table S3. EGFR Mutation Prediction Results Base Classifiers

Feature Selection	Classifier	Train				Test			
		Acc.	Sens.	Spec.	AUC	Acc	Sens.	Spec.	AUC
**ReliefF (15 features)**	**SVM**	**0,83**	**0,79**	**0,86**	**0,88**	**0,76**	**0,66**	**0,78**	**0,68**
ReliefF (10 features)	RF	0,99	0,99	1,00	0,99	0,76	0,41	0,82	0,67
ReliefF (15 features)	nnet	0,77	0,59	0,91	0,78	0,76	0,58	0,79	0,67
ReliefF (5 features)	RF	0,99	0,99	1,00	0,99	0,77	0,50	0,82	0,64
ReliefF (20 features)	RF	0,99	0,99	1,00	0,99	0,73	0,16	0,83	0,63
ReliefF (20 features)	SVM	0,85	0,81	0,89	0,91	0,68	0,66	0,69	0,63
ReliefF (5 features)	nnet	0,74	0,60	0,86	0,80	0,71	0,50	0,75	0,60
ReliefF (5 features)	SVM	0,70	0,48	0,87	0,67	0,79	0,25	0,89	0,59
ReliefF (15 features)	RF	1,00	1,00	1,00	1,00	0,72	0,25	0,80	0,57
MW (5 features)	gbm	0,97	0,97	0,98	0,99	0,68	0,16	0,78	0,53
ReliefF (15 features)	gbm	0,99	0,99	1,00	1,00	0,72	0,25	0,80	0,52
ReliefF (20 features)	nnet	0,77	0,74	0,81	0,79	0,55	0,50	0,57	0,52
MW (20 features)	SVM	0,97	0,98	0,97	0,98	0,71	0,25	0,79	0,52
ReliefF (5 features)	gbm	0,97	0,95	0,98	0,99	0,71	0,33	0,78	0,51
ReliefF (10 features)	nnet	0,77	0,63	0,88	0,80	0,63	0,16	0,72	0,51

Table S4. KRAS Mutation Prediction Results, Base Classifiers

Feature Selection	Classifier	Train				Test			
		Acc.	Sens.	Spec.	AUC	Acc.	Sens.	Spec.	AUC
**ReliefF (5 features)**	**SVM**	**0,67**	**0,39**	**0,89**	**0,73**	**0,70**	**0,35**	0,81	**0,65**
MW (15 features)	SVM	0,85	0,77	0,91	0,92	0,64	0,40	0,72	0,64
ReliefF (5 features)	gbm	0,99	0,99	1,00	0,99	0,64	0,60	0,65	0,63
ReliefF (20 features)	gbm	0,99	0,99	1,00	0,99	0,63	0,50	0,67	0,63
MW (10 features)	SVM	0,82	0,73	0,89	0,90	0,64	0,45	0,70	0,63
MW (20 features)	SVM	0,90	0,85	0,95	0,96	0,71	0,40	0,81	0,63
ReliefF (15 features)	SVM	0,78	0,70	0,85	0,85	0,67	0,45	0,75	0,62
MW (5 features)	SVM	0,73	0,48	0,91	0,83	0,71	0,35	0,83	0,62
MW (15 features)	gbm	1,00	1,00	1,00	1,00	0,67	0,40	0,77	0,62
ReliefF (15 features)	RF	1,00	1,00	1,00	1,00	0,62	0,40	0,70	0,60
MW (10 features)	gbm	1,00	1,00	1,00	1,00	0,67	0,40	0,76	0,59
MW (20 features)	gbm	1,00	1,00	1,00	1,00	0,69	0,30	0,81	0,59
ReliefF (10 features)	gbm	0,99	0,99	1,00	1,00	0,55	0,35	0,62	0,57
MW (5 features)	nnet	0,81	0,68	0,92	0,85	0,57	0,45	0,60	0,56

Table S5. EGFR Mutation Best Results, CNNs

Model	Optimizer	Learning Rate	Epochs	Train				Test			
				Acc.	Sens.	Spec.	AUC	Acc.	Sens.	Spec.	AUC
**Arch. 4**	**SGD**	**0,0005**	**30**	0,947	0,900	0,965	0,952	**0,800**	**0,667**	**0,846**	**0,846**
**Arch. 6**	**SGD**	**0,0005**	**30**	0,947	0,825	0,991	0,938	**0,771**	**0,222**	**0,961**	**0,752**
Arch. 1	SGD	0,01	8	0,555	0,950	0,416	0,936	0,400	1,000	0,192	0,688
Arch. 6	SGD	0,01	10	0,889	0,675	0,965	0,916	0,657	0,666	0,654	0,675
Arch. 3	SGD	0,01	7	0,791	0,900	0,752	0,916	0,543	0,778	0,461	0,671
Arch. 4	SGD	0,01	10	0,849	0,425	1,000	0,825	0,543	0,778	0,461	0,628
Arch. 1	SGD	0,01	30	0,706	0,350	0,832	0,724	0,514	0,778	0,423	0,623
Arch. 2	SGD	0,01	30	0,565	0,162	0,849	0,652	0,542	0,667	0,538	0,571
Arch. 4	SGD	0,01	20	0,591	0,200	0,867	0,519	0,600	0,444	0,654	0,559

Table S6. KRAS Mutation Best Results, CNNs

Model	Optimizer	Learning Rate	Epochs	Train				Test			
				Acc.	Sens.	Spec.	AUC	Acc.	Sens.	Spec.	AUC
**Arch. 1**	**SGD**	**0,01**	**60**	**0,698**	**0,045**	**0,971**	**0,602**	**0,667**	**0,000**	**1,000**	**0,739**
Arch. 6	Adam	0,005	10	0,329	0,795	0,133	0,491	0,333	1,000	0,000	0,607
Arch. 6	Adam	0,001	10	0,704	0,000	1,000	0,538	0,667	0,000	1,000	0,593
**Arch. 1**	**Adam**	**0,005**	**15**	**0,751**	**0,204**	**0,981**	**0,588**	**0,722**	**0,250**	**0,958**	**0,566**
Arch. 1	SGD	0,01	90	0,684	0,159	0,905	0,588	0,667	0,000	1,000	0,555
**Arch. 1**	**SGD**	**0,01**	**10**	**0,637**	**0,659**	**0,628**	**0,700**	**0,555**	**0,667**	**0,500**	**0,531**
